# The physiological characteristics of the basal microvilli microvessels in pancreatic cancers

**DOI:** 10.1002/cam4.3177

**Published:** 2020-06-02

**Authors:** Lixiang Ma, Xu Han, Jichun Gu, Ji Li, Wenhui Lou, Chen Jin, Hexige Saiyin

**Affiliations:** ^1^ Department of Anatomy, Histology & Embryology School of Medical Sciences Fudan University Shanghai China; ^2^ General Surgery Department Zhongshan Hospital Fudan University Shanghai China; ^3^ Department of Pancreatic Surgery Huashan Hospital Fudan University Shanghai China; ^4^ The State Key Laboratory of Genetic Engineering Institute of Genetics School of Life Sciences Fudan University Shanghai China

## Abstract

Pancreatic cancer (PC) is a highly lethal tumor with controversial high glucose uptake and hypomicrovascularity, and the hypomicrovasculature, which is considered to have poor perfusion, blocks the delivery of drugs to tumors. The preferential existence of a novel endothelial projection with trafficking vesicles in PCs, referring to basal microvilli, was described previously. However, the perfusion and nutrients delivering status of the basal microvilli microvessels are unknown. Here, we used the perfusion of fluorescently labeled CD31 antibody, lectin, and 2‐NBDG to autochthonous PC‐bearing mice, immunostaining, probe‐based confocal laser endoscopy and three‐dimensional (3D) reconstruction to study the nutrient trafficking, and perfusion status of the basal microvilli microvasculature in PC. Our data showed that the coperfusion of lectin and CD31 is an efficient way to show the microcirculation in most healthy organs. However, coperfusion with lectin and CD31 is inefficient for showing the microcirculation in PCs compared with that in healthy organs and immunostaining. This method does not reflect the nutrient trafficking status in the microvessels, especially in basal microvilli microvessels of PCs. In basal microvilli microvessels that were poorly labeled by lectin, we observed large vesicle‐like structures with 2‐NBDG preferentially located at the base of the basal microvilli or in basal microvilli, and there were long filopodia on the luminal surface of the human PC microvasculature. Our observations suggest that the PC microvasculature, especially basal microvilli microvessels, is well perfused and might be highly efficient in the trafficking of glucose or other nutrients, indicating that macropinocytosis might participate in the nutrient trafficking.

## Graphical abstract

Previously, we described the preferential existence of a novel endothelial projection with trafficking vesicles in PCs, referring to basal microvilli. Here, we perfused multiple endothelial markers and nutrients to autochthonous PC‐bearing mice to study the nutrient trafficking and perfusion status of the basal microvilli microvasculature. Our observations suggest that the PC microvasculature, especially basal microvilli microvessels, is well perfused and might be highly efficient in the trafficking of glucose or other nutrients, indicating that macropinocytosis might be the main method of nutrient trafficking.

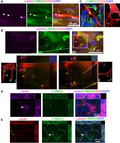

## 1 INTRODUCTION

Pancreatic cancers (PCs) are a highly lethal solid tumor with controversial hypomicrovascularity, high glucose uptake, high interstitial pressure, and abundant desmoplastic stroma.^1^, ^2^ The hypomicrovasculature in PC is described as poorly perfused, compressed, and inefficient in terms of nutrient exchange and drug delivery.^3^, ^4^, ^5^, ^6^ These characteristics of microvessels in PC are controversial given its high metabolism and efficient glucose and albumin uptake but consistent with inefficient drug delivery.^7^, ^8^, ^9^ Epithelial projections, such as microvilli in the intestine and kidney, are the most efficient way to increase nutrient or waste exchange in organs.^10^ A novel endothelial projection with nutrient trafficking vesicles, referring to basal microvilli, ubiquitously present on the basal surface of the PC microvasculature, and its abundance correlated with patients' PET‐CT scores.^11^ The presence of basal microvilli might explain why glucose and albumin easily reach PCs, but drugs do not. However, the physiology of the basal microvilli microvasculature, including blood flow and nutrients trafficking, is unknown.

Microcirculation composed of the arteriole, capillary network, and postcapillary vein supports oxygen delivery, nutrient exchange, and removal of waste and controls blood flow, hemodynamics, coagulation, inflammation, immune surveillance and metastasis of cancer cells.^12^, ^13^ The microvasculature differs in size and function across different organs, and there are even different segments in the same organ and tumors.^13^, ^14^ Intravital microscopy (IVM) is a tool for investigating microvascular dynamics in vivo.^13^, ^14^ The dense stroma and rare microvascularity in PCs make the PC microcirculation challenging to visualize or analyze in vivo. Based on the specific molecules expressed on endothelial cells, immunostaining with endothelial markers is utilized to visualize the microvasculature structure. Immunostaining in histological slides lacks depth and width and makes it challenging to analyze pathophysiology.^15^ Intravital injection of fluorescently labeled lectins and CD31 antibody or inks has been used to avoid damage to endothelial antigens during the preparation of histological samples and show the pathophysiology of microvessels.^16^, ^17^ Intravital injections of fluorescently labeled lectins and CD31 antibody and thick section screening reveal the varied morphology of microvessels across different organs pathophysiological circumstances, such as liver, lung, kidney, and brain, and reflect endothelial functions such as permeability, endocytosis, and transportation as well.^13^, ^16^, ^18^, ^19^, ^20^, ^21^ Cooperation with other dyes such as 2‐NBDG and DAPI could additionally contribute to evaluation of the permeability of capillaries and its relationship with surrounding tissues in murine models.^22^ Thus, a combination of different labeling dyes or antibodies and nutrient analogs might have the potential to reveal blood flow dynamics and nutrient trafficking in basal microvilli microvessels.

Basal microvilli microvasculature exists in murine autochthonous PCs that harbor the *KRAS* mutation but not xenograft or orthotopic tumors.^11^, ^23^ These findings provide us with a limited tool to study the nutrient trafficking and blood flow of basal microvilli microvessels in PCs. To reveal the blood flow dynamics and nutrient exchange of the microcirculation in PCs, we perfused the circulation with several endothelial markers and 2‐NBDG and used immunostaining and high‐resolution confocal microscopy as well as imaging of human pancreatic cancer by probe‐based confocal laser endoscopy. Our multiple approaches reveal that perfused labeling with lectin or endothelial markers does not match with the nutrient trafficking and perfusion status in the murine PC microvasculature, especially the microvasculature with basal microvilli. This finding shows that the basal microvilli microvessels in pancreatic cancer might have a strong ability to traffic nutrients and have efficient blood flow.

## 2 MATERIALS AND METHODS

### 2.1 Ethics statements

All genetically engineered mouse models (GEMMs) and C57BL/6 were breed and maintained in standard animal facilities, and the Institutional Animal Care and Use Committee of Fudan University supervised and approved all animal procedures. The ethics committee of Huashan Hospital of Fudan University approved the human ethics.

### 2.2 Breeding of GEMMs pancreatic cancer mice

Eight to 10‐week‐old C57BL/6 (male) mice were purchased from the Animal Center of Fudan University. The breeding of GEMMs was described previously.^11^ Briefly, to breed *LSL‐Kras^G12D^; LSL‐Trp53^R172H/+^; Ink4^flox/+^; Ptf1/p48‐Cre* (KPIC) mice, *LSL‐Kras^G12D^; LSL‐Trp53^R172H/+^* mice were crossed with *Ink4^flox/flox^; Ptf1/p48‐Cre* (IC) mice. To breed *LSL‐Kras^G12D^; Ink4^flox/+^; Ptf1/p48‐Cre* (KIC) mice, *Ink4^flox/flox^; Ptf1/p48‐Cr* (IC) mice were crossed with *LSL‐Kras^G12D^; Ink4^flox/flox^* mice. Genotyping was done by following the standard protocols of providers (Jackson Lab). KPC mice tissues were provided by DrTuveson lab.

### 2.3 Perfusion of tumor‐bearing mice with Lectin‐Alexa 633 and 2‐NBDG‐Alexa 488

Lectin‐Alexa 633, CD31‐FITC antibody, or 2‐NBDG‐Alexa 488 was dissolved in 0.01‐mM phosphate‐buffered saline (PBS). Tumor‐bearing GEMMs mice including four 8‐week‐old KIC mice, two 5‐month‐old KPC mice, and three 12‐week‐old KPIC mice or five C57BL/6 were deeply anesthetized by isoflurane, and Lectin‐Alexa 633 (0.5 mg/mL), CD31‐FITC, or 2‐NBDG‐Alexa 488 (0.5 mg/mL) was perfused from the left heart ventricle of the mice with a TB syringe. Ten minutes after injection, the mice were sacrificed, and the tissues were fixed with 4% paraformaldehyde (PFA) for microscopy analyses.

### 2.4 H&E and immunofluorescent staining

Fresh tissue samples were fixed in freshly prepared 4% PFA overnight and cryoprotected in 30% sucrose/PBS buffer. Thick slides, including normal and tumor tissues, were stained and scanned with confocal microscopy, as previously described.^11^ The Z‐stack scanned images were 3D reconstructed by Zen 2012 software (Zeiss) or Fiji ImageJ software (NIH). Tissue staining was performed as previously described.^11^ The following antibodies were used in immunohistochemistry and immunofluorescent staining: CD31‐FITC (Rat, BD; clonal number, Clone MEC 13.3), CD34 (mouse monoclonal, Abcam, 8536; rabbit polyclonal antibody; Abcam; EP373Y; Rat Anti‐Mouse CD34 Clone RAM34), anti‐rabbit Alexa Fluor 555 conjugated immunoglobulin G (Donkey; Life Technologies), DAPI (Sigma), 2‐NBDG and Lectin‐Alexa 633 (Sigma), VEGFR2/KDR/Flk‐1 Antibody (Goat, R&D,CAT# AF357), Phospho‐KDR/FLK1‐Y996 Antibody (Rabbit, Epitomics, Epitomics‐T3718). Finally, multiple organs, including heart coupled with lung, liver, spleen, pancreas, kidney, brain, and skeleton, were excised and kept in 4% PFA.

### 2.5 Videos of the microcirculation in the human pancreas and pancreatic cancer

Dr Jin Chen obtained clinical videos by probe‐based confocal laser endoscopy (Cellvizio) in clinics.

### 2.6 Human tissue culture

Fresh surgical samples were collected and cultured in 10% fetal bovine serum (Gibco) and high glucose Dulbecco's modified Eagle medium (glucose, 4500 mg/L; Gibco). After culturing for 6 hours, we fixed the tissue in 4% PFA overnight and immunostained with the CD34 antibody.

### 2.7 Image analysis

We used IMARIS.9.5 (Oxford Fundamental, UK) and Zen 2012 Black Edition (Zeiss) to analyze and process images. The filament tool used to show basal microvilli; the spot tool used to show 2‐NBDG; the surface tool used to show microvasculature surface.

## 3 RESULTS

### 3.1 The perfused staining of Lectin‐Alexa 633 and CD31‐FITC efficiently showed the microcirculation of healthy murine organs

**FIGURE 1:**
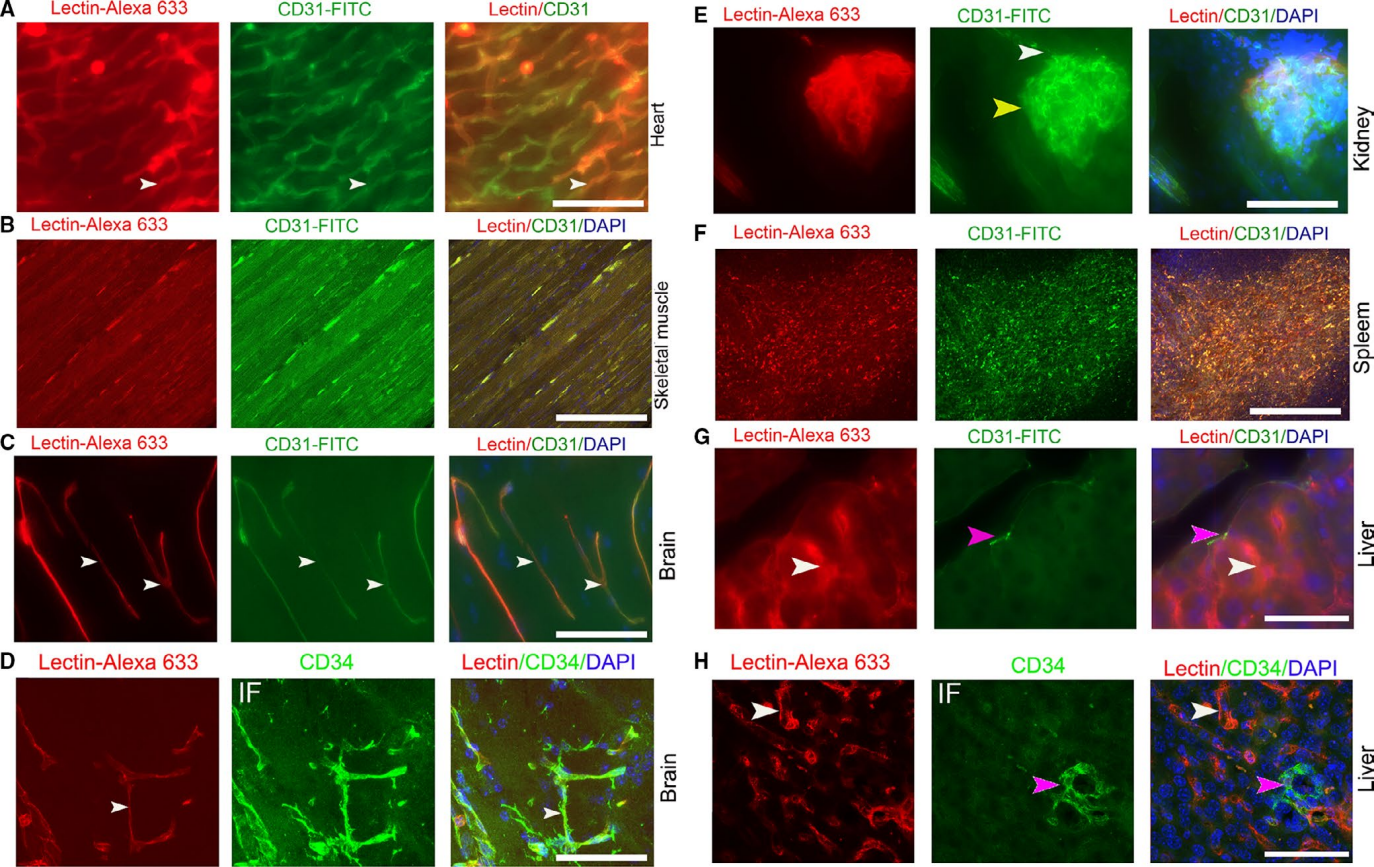
The perfused staining of Lectin‐Alexa 633 and CD31‐FITC in normal murine organs. (A, B, C) Images of coperfused staining with CD31‐FITC and Lectin‐Alexa 633 in the heart, skeletal muscles, and brain of B6/C57 mice (white arrows, typical slender microvessels). (D) Comparison of coperfused staining of Lectin‐Alexa 633 in the brain with immunostaining with a CD34 antibody (white arrows, typical slender microvessels). (E) Images of coperfused staining with CD31‐FITC and Lectin‐Alexa 633 in the kidney (yellow arrow, glomerulus; white arrow, afferent artery). (F, G) Images of coperfused staining with CD31‐FITC and Lectin‐Alexa 633 in spleen and liver (pink arrow, central vein; white arrow, sinusoid endothelial cell). (H) Comparison of coperfused staining of lectin with CD34 antibody immunostaining in livers (white arrow, sinusoid endothelial). Mice, n = 3. IF, immunofluorescent staining. Scale bar, 50 µm

The endothelial cells in healthy organs and tumors ubiquitously expressed CD31 and CD34 molecules and lectin receptor.^24^, ^25^, ^26^ To establish an efficient method to show the perfusion of the microcirculation in organs, we perfused C57BL/6 mice with Lectin‐Alexa 633 and CD31‐FITC antibody from the left ventricle, a systemic delivery method. Scanning images showed that both Lectin‐Alexa 633 and CD31‐FITC antibody efficiently labeled the organs with typical continuous capillaries, including the heart, skeletal muscles, and brain; the microvasculature was shown as a slender and smooth structure that met the characteristics of continuous capillaries with good perfusion (Figure 1A‐C). We selected the brain for staining with another CD34 antibody. In our previous works, we found that immunostaining with the CD34 antibody in a thick section completely overlapped with CD31 antibody staining in multiple tissues. We found that the immunostaining results with a CD34 antibody entirely overlapped with that of CD31 in the brain (Figure 1D).

Contrary to immunostaining with a CD31 antibody,^27^ we observed that both Lectin‐Alexa 633 and CD31‐FITC antibody well characterized the afferent arteriole of Bowman's capsule and the capillary network of the renal corpuscle. These results are consistent with other observation. However, the efferent arteriole and its subsequent capillary beds were inefficiently labeled by both Lectin‐Alexa 633 and CD31‐FITC antibody (Figure 1E). In organs with sinusoid capillaries, including spleen and liver, the labeling of CD31 and lectin matched each other in the spleen; CD31 labeled the central vein and other larger vessels but did not label the sinusoidal endothelium in livers, and lectin labeled both central veins and all endothelial cells of the sinusoid (Figure 1F,G). We also used the CD34 antibody to stain the perfused microvessels in the liver. The staining results are consistent with the perfused labeling of the CD31 antibody (Figure 1H). The coperfusion of lectin and CD31 is an efficient way to label the microvasculature in most organs except the kidney.

### 3.2 The basal microvilli microvasculature in autochthonous PCs resembles the human basal microvilli microvasculature

**FIGURE 2:**
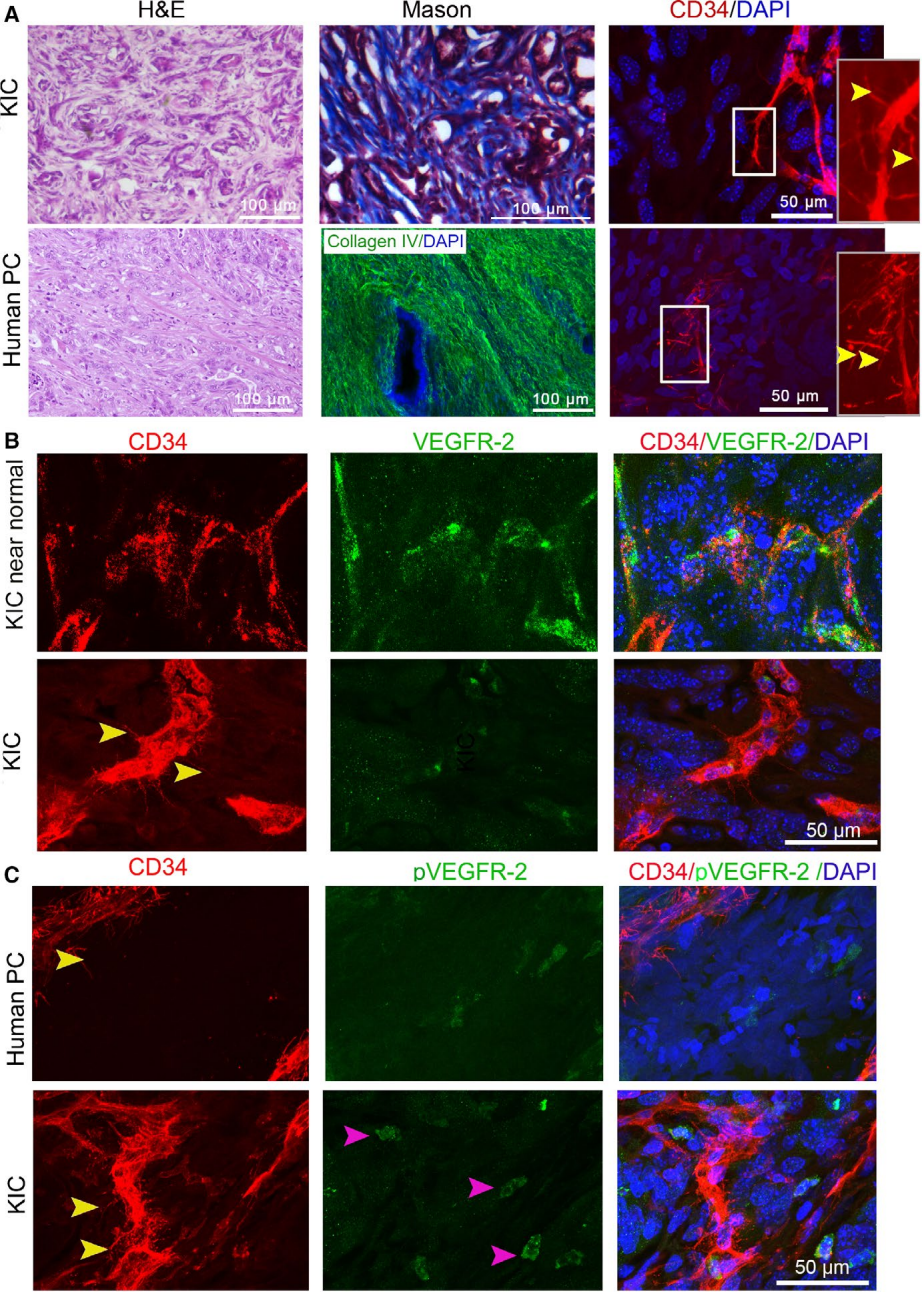
Basal microvilli microvasculature in autochthonous PC in KIC resembles human basal microvilli microvasculature. (A) Comparison of the histology, stroma, and basal microvilli microvessels of the PC in KIC mice with human PC (yellow arrows, basal microvilli). A collagen IV antibody (green) stains human stroma. The inner panel, magnified region. (B) Comparison of VEGFR2 expression patterns in the basal microvilli microvessels of the PC in KIC with that in the near‐normal pancreatic tissue of KIC mouse (yellow arrows, basal microvilli). (C) Comparison of phospho‐VEGFR2^Y996^ (pVEGFR2^Y996^) expression levels in the basal microvilli microvessels of the PC in KIC with that in human PC (yellow arrows, basal microvilli). Mice, n = 2

**FIGURE 3:**
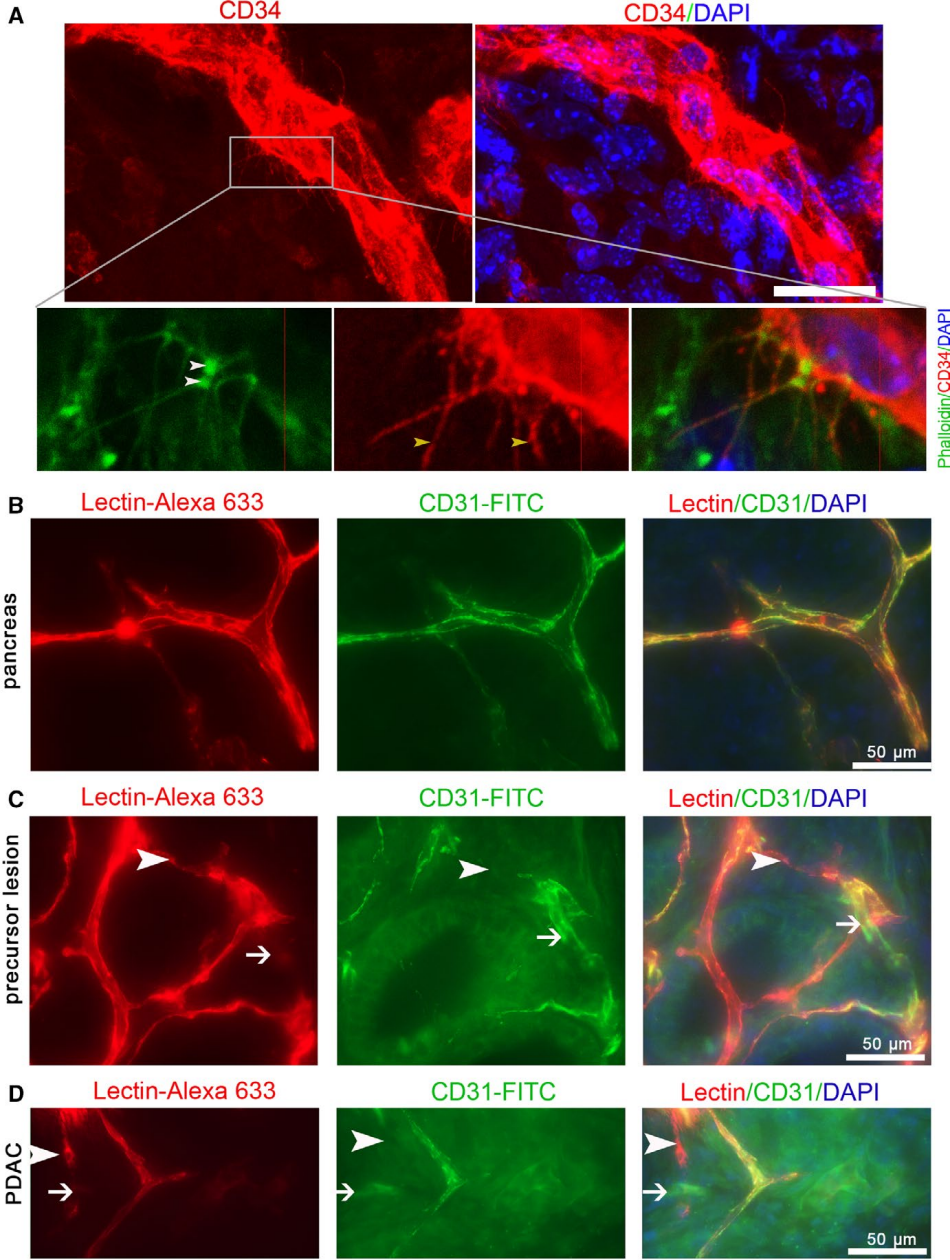
Perfused staining with Lectin‐Alexa 633 or CD31‐FITC is inefficient for labeling the microvasculature in precursor lesions and pancreatic cancer of KIC mice. (A) Phalloidin staining shows the actin cytoskeleton of the basal microvilli in the PC of KIC. (B, C, D) Comparison of the perfused staining of CD31‐FITC with that of Lectin‐Alexa 633 in the normal pancreas, precursor lesion, and PC of KIC mice (white arrows and arrowheads, partially matched parts). n = 2

The autochthonous PCs of GEMMs that harbor *KRAS* and *TP53* or *CDKN2A* or *SMAD4* mutations contain abundant dense stroma and hypomicrovascularity.^28^, ^29^, ^30^ The pathophysiology and drug response of autochthonous PCs in GEMMs are near to human PC.^31^ The autochthonous PCs in KPC and KPIC present basal microvilli.^11^, ^23^ KIC mouse that harbor *KRAS* and *Ink4* mutation form a highly lethal PC with near 2‐month survival, and the PCs in KIC present hypomicrovascularity and rich stroma.^29^ Thus, it is possible that the microvasculature of autochthonous PC in KIC also present basal microvilli. To see if the microvasculature in the PCs of KIC presents basal microvilli, we have stained the PCs of KIC with a CD34 antibody. Similar to the microvasculature of human pancreatic cancers, autochthonous KPC, and KPIC tumors,^11^, ^23^ the microvasculature in KIC tumors also presents basal microvilli (Figure 2A). Consistent with the characteristics of human basal microvilli microvasculature,^11^ we observed that the basal microvilli microvasculature in both KPC and KIC tumors has a lower level of VEGFR2 and pVEGFR2 (Y966) when compared to the microvessels in the near‐normal tissue (Figure 2B,C; Figure S1A,B). The cytoskeleton of the basal microvilli in human PC contains actin filaments.^11^ To observe the cytoskeleton of the basal microvilli in KIC PC, we stained the basal microvilli with a CD34 antibody and the cytoskeleton with phalloidin. The results showed that the cytoskeleton of basal microvilli in KIC is actin based (Figure 3A). These data support the notion that the basal microvilli microvasculature in KIC and KPC tumors structurally and functionally resembles the human basal microvilli microvasculature, indicating that KIC, KPC, and KPIC are suitable models for exploring the physiology of basal microvilli microvessels.

### 3.3 Perfused staining with Lectin‐Alexa 633 and CD31‐FITC is inefficient for showing the basal microvilli microvasculature of murine autochthonous PCs

**FIGURE 4:**
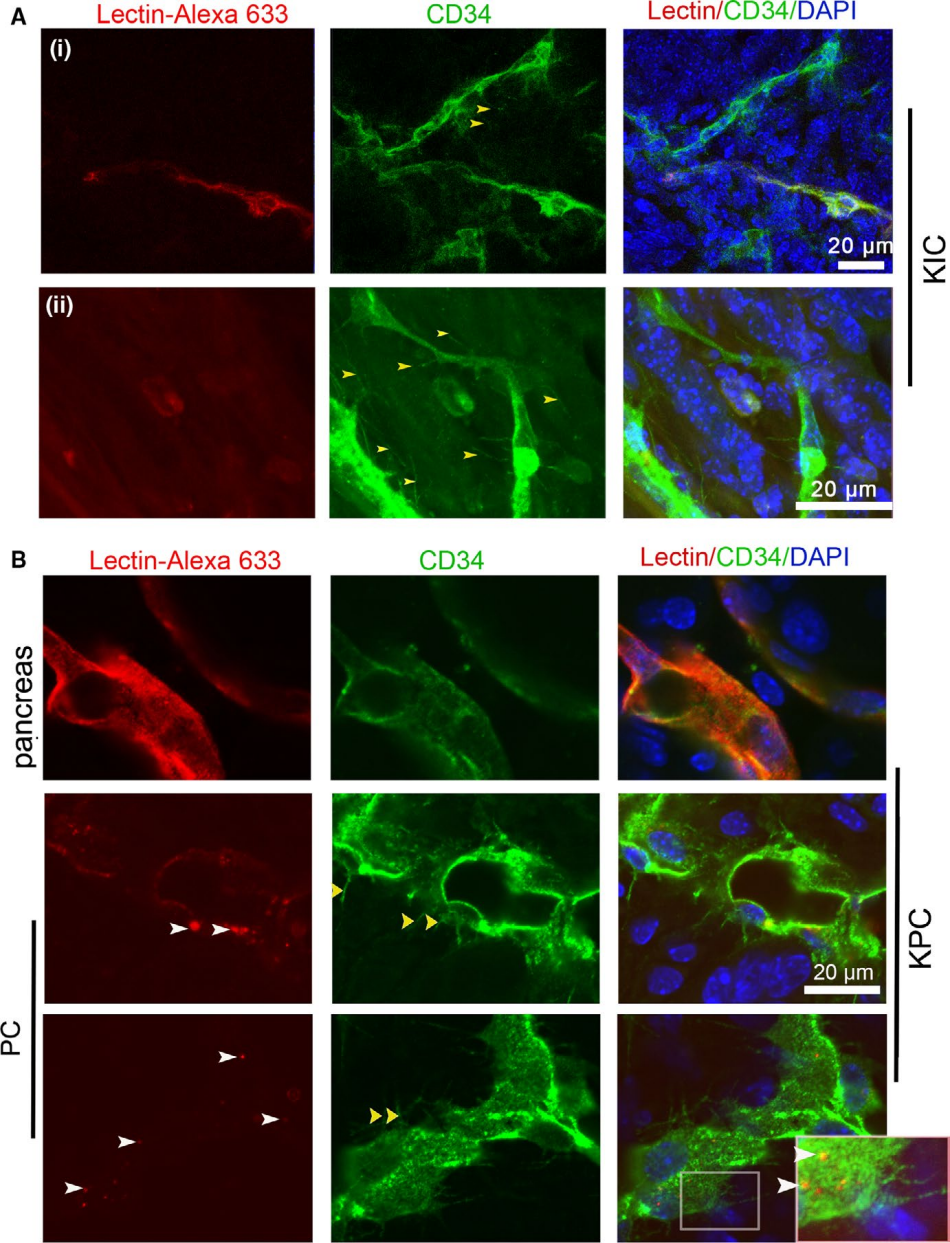
Perfused staining with Lectin‐Alexa 633 and CD31‐FITC is inefficient for showing the basal microvilli microvasculature in autochthonous PCs. (A) Comparison of the perfused staining of Lectin‐Alexa 633 with CD34 antibody immunostaining in the tumors of KIC mice (yellow arrows, basal microvilli; white arrows, lectin). (B) Comparison of the perfused staining of Lectin‐Alexa 633 with CD34 antibody immunostaining in the tumors of KPC mice (yellow arrows, basal microvilli; white arrows, lectin). Boxed area, the magnified region

To characterize the basal microvilli microcirculation, we selected tumor‐bearing KIC mice and perfused them with a mixture of Lectin‐Alexa 633 and CD31‐FITC. Our scanning data of thick sections showed that the perfused labeling of Lectin‐Alexa 633 in healthy pancreas tissues of KIC mice completely overlapped with that of CD31‐FITC and efficiently showed the microvasculature (Figure 3B). However, the perfused labeling of Lectin‐Alexa 633 in the precursor lesions partially overlapped with that of CD31‐FITC, the overlapping decreased from the precursor lesions to tumor regions, and some microvessels labeled with Lectin‐Alexa 633 were absent with CD31‐FITC labeling and *vice versa* (Figure 3C,D). To further test if some microvessels in KIC tumors are completely unlabeled by Lectin‐Alexa 633, we stained the tissues perfused by Lectin‐Alexa 633 with a CD34 antibody. We found that perfused labeling with Lectin‐Alexa 633 did not show a significant number of microvessels in PCs of KIC (Figure 4Ai), especially the microvessels that present basal microvilli (Figure 4Aii). To determine if limited or nonbinding of the lectin to the endothelial cells in PCs is common, we perfused tumor‐bearing KPC mice with Lectin‐Alexa 633. After immunostaining with a CD34 antibody, we also constructed entire microvessels with high‐resolution imaging confocal microscopy. Contrary to microvessels in the healthy pancreas and similar to KIC tumors, we observed that the lumen of basal microvilli microvessels contains a limited amount of lectin and is visible as scattered dots (Figure 4B). These data suggest that the basal microvilli microvessels in the PC of GEMMs have blood flow.

### 3.4 The basal microvilli microvessels in human PCs have efficient blood flow and might depend on micropinocytosis for nutrient trafficking

**FIGURE 5:**
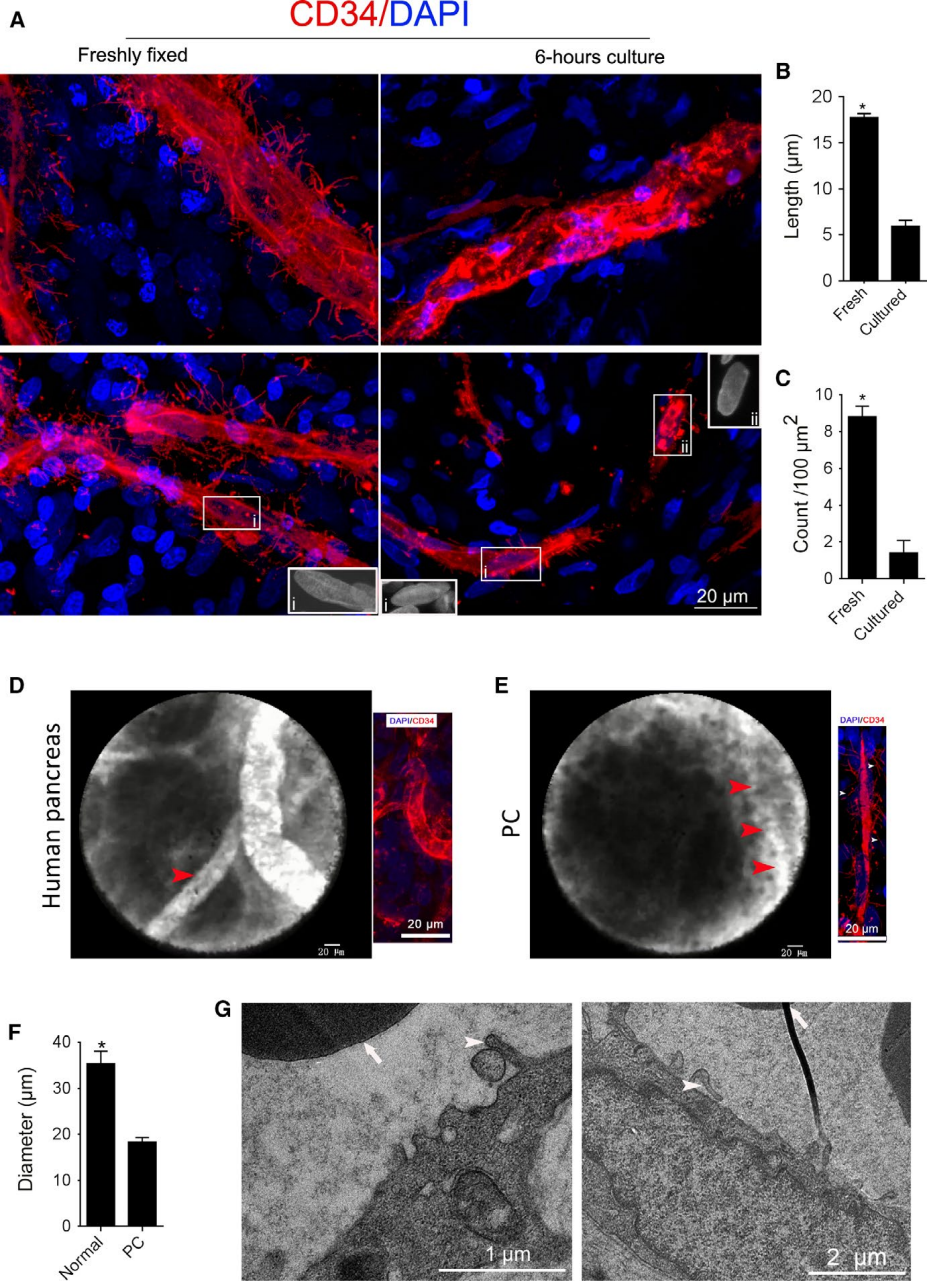
The basal microvilli microvessels in human PCs have efficient blood flow and might depend on micropinocytosis for nutrient trafficking. (A) The representative images of the basal microvilli of freshly fixed PC with 6‐hour‐cultured PC tissues from the same patient. The morphology of endothelial nucleus, inner panel. (B, C) Comparison of the basal microvilli length and density of freshly fixed PC with 6‐hour‐cultured PC tissues from the same patient. Data show mean ± SE. Unpaired student's *t* test, *<0.05. (D, E) Comparison of the blood flow of the microcirculation in the healthy human pancreas with PC. Probe‐based laser confocal endomicroscopy images and video (red arrow, microvessel; white arrows, basal microvilli). Healthy pancreas, n = 2; PC, n = 5. See Videos [Link], [Link] . (F) Comparison of the diameters of the microcirculation in the healthy human pancreas with PC. Data show mean ± SE. Unpaired student's *t* test, *<0.05. (G) TEM images of the luminal surface in the PC microvasculature in human PC (white arrows, red blood cells; arrowheads, endothelial projections, or filopodia)

To observe the relationship of blood flow with basal microvilli, we cultured human PC tissues with high glucose uptake medium (PET‐CT SUVmax, 8.9) in dishes. By comparing it with its freshly fixed counterpart, we found that the basal microvilli in the cultured PC tissues became shorter and thinner compared to the freshly fixed tissue after surgery (Figure 5A‐C). This observation indicated that blood flow in the microvasculature might be necessary for the growth of basal microvilli. To observe the characteristics of blood flow in the microvasculature of PCs, we analyzed videos of the human pancreas and PC microvasculature taken by probe‐based confocal laser endoscopy (Cellvizio) in the clinic. We observed that the diameters of microvessels were thinner than those of a healthy pancreas (Figure 5D‐F), and the fluorescein flow in the PC microvasculature was observably quicker compared to the flow in the healthy pancreas (Video S1A,B). Additionally, we observed a significant amount of fluorescein dots in the tumor milieu and active tissue fluid flow but did not observe fluorescein dots in normal pancreas (Figure 5E,D; Video S1A,B). To determine whether the luminal surface of the endothelial cells in PCs has characteristics that facilitate macropinocytosis, we evaluated TEM images of PC microvessels and observed multiple longer projections on the luminal surface of microvessels, resembling the macropinocytic filopodia (Figure 5G). This observation indicated that the quick blood flow in the PC microvasculature might be a way to overcome the high interstitial pressure in the stroma, and macropinocytosis might be a way to exchange nutrients and waste.

### 3.5 The perfused staining of lectin does not reflect nutrient trafficking in the basal microvilli microvasculature of murine PCs

**FIGURE 6:**
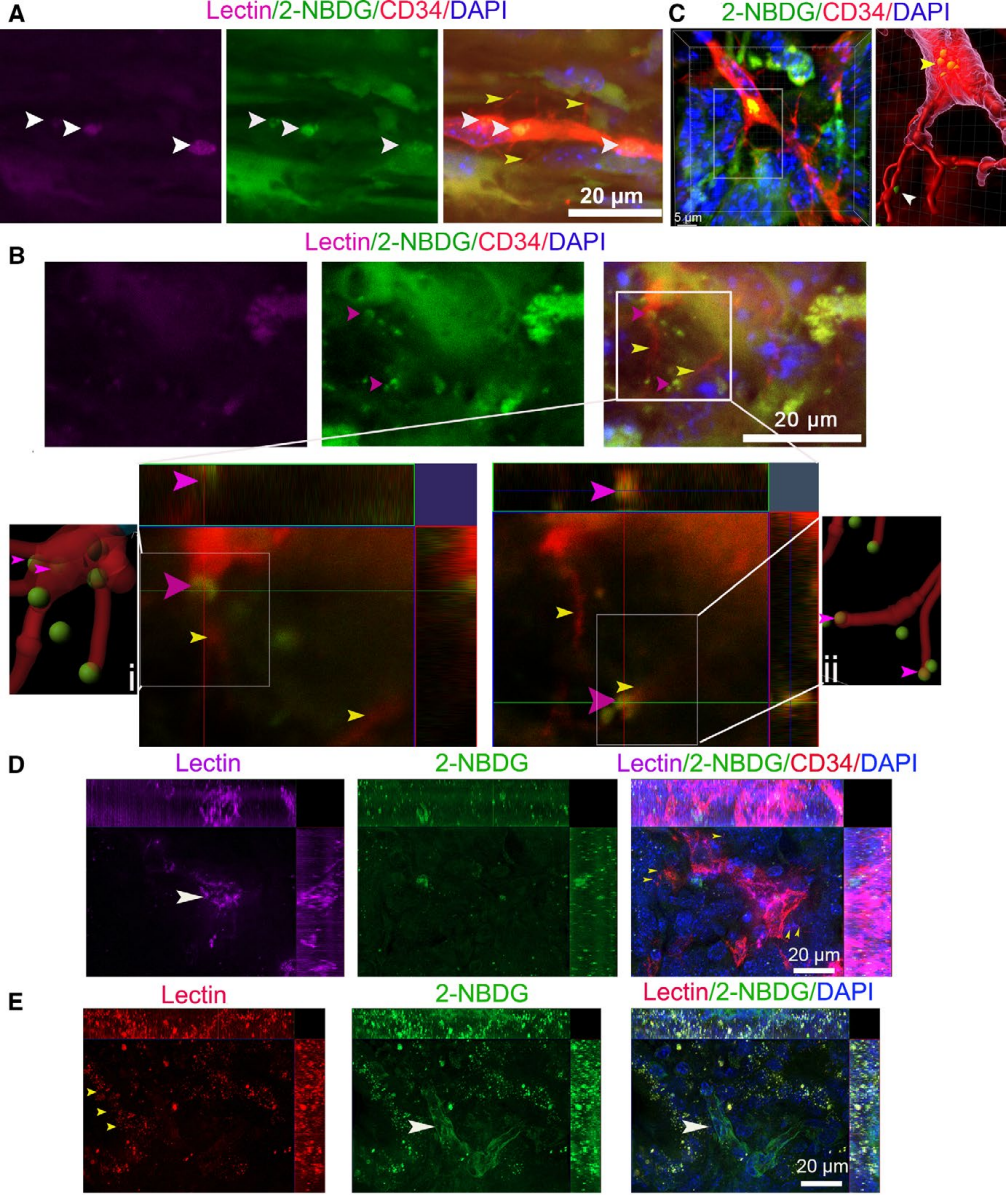
Perfused staining results with lectin do not reflect the nutrient trafficking in PCs. (A0 PC images in KIC mice coperfused with Lectin‐Alexa 633 and 2‐NBDG were stained by CD34 antibody (yellow arrows, basal microvilli; white arrows, 2‐NBDG). (B) Images of 2‐NBDG distribution in the basal microvilli microvasculature of KIC mice coperfused with Lectin‐Alexa 633 and 2‐NBDG. The two images in lower panels show magnification of the boxed region in the upper panel (at different Z‐layers). IMARIS spots and filament analyses showed that 2‐NBDG exists in basal microvilli or binds with basal microvilli (pink arrows, 2‐NBDG‐containing vesicle‐like structure; yellow arrows, basal microvilli). (C) A typical 2‐NBDG vesicle‐like structure present at the base of the basal microvilli in the microvasculature lumen of KC mice coperfused with Lectin‐Alexa 633 and 2‐NBDG and stained by CD34 antibody Right panel is the image processed by Imaris 9.5 (yellow arrow, basal 2‐NBDG vesicle‐like structure; white arrow, 2‐NBDG in basal microvilli). (D) Images of CD34 antibody immunostaining in the PC of a KPIC mouse coperfused with 2‐NBDG and Lectin‐Alexa 633 showed that Lectin‐Alexa 633 partially labeled the microvasculature of basal microvilli (white arrow, microvessel; yellow arrows, basal microvilli). (E) Images of the PC in KPIC mice co‐perfused with 2‐NBDG and Lectin‐Alexa 633 show that the microvessels are poorly labeled by Lectin‐Alexa 633 but contain decent levels of 2‐NBDG (white arrow, microvessels; pink arrows, tumor cells; yellow arrows, lectin dots in neoplastic cells)

PCs have a strong ability to take up glucose.^32^, ^33^ We found that the basal microvilli contain GLUT‐1+ vesicles, and their abundance is related to high glucose uptake ability in human PCs.^11^ Thus, we hypothesized that basal microvilli microvessels that are incompletely labeled with Lectin‐Alexa 633 or CD31‐FITC are efficient in nutrient trafficking. To test if basal microvilli microvessels have glucose trafficking ability, we perfused tumor‐bearing KIC mice with Lectin‐Alexa 633 and 2‐NBDG, a glucose analog. In higher resolution images, we observed multiple bunches of 2‐NBDG in the lumen of tumor basal microvilli microvasculature that were inefficiently delineated by Lectin‐Alexa 633 (Figure 6A). To determine if the basal microvilli that protrude from microvessels facilitate 2‐NBDG trafficking, we scanned the basal microvilli with higher resolution, and then performed analysis with Imaris software 9.5. Surprisingly, we observed that the basal microvilli in KIC contain many vesicles‐like structures that contain 2‐NBDG (Figure 6B,C). Additionally, we noticed that most 2‐NBDG bunches were preferentially located at the base of the basal microvilli in the vascular lumen (Figure 6C). We also counted the 2‐NBDG in the basal microvilli, and found 30% (9/30) basal microvilli contain 2‐NBDG. These observations further support that basal microvilli facilitate glucose trafficking.

Wild‐type p53 expression decreases glucose uptake, whereas mutation or deletion of p53 strengthens the glucose uptake in PC.^34^, ^35^ In total, 60%‐70% of human PCs have p53 mutation or deletion.^36^ To see if the nutrient trafficking of basal microvilli microvessels with inefficient lectin binding was universal in PCs, we selected a tumor‐bearing KPIC mouse that harbors a p53 mutation. KPIC forms a typical ductal adenocarcinoma with high glucose uptake and contains abundant dense stroma.^23^ We perfused KPIC mice with Lectin‐Alexa 633 and the 2‐NBDG mixture. Similar to the perfused staining in KIC and KPC tumors, we observed that the microvessel segments with basal microvilli in KPIC tumors were poorly labeled by lectin (Figure 6D). We found that the microvessel sections with low lectin binding contained vast amounts of 2‐NBDG in the microvascular lumen, and the surrounding tumor cells accumulated enormous amounts of 2‐NBDG and lectin dots in tumor cells (Figure 6D,E). However, the microvessels with lectin binding did not accumulate 2‐NBDG in the lumen, and the accumulation of 2‐NBDG in surrounding tumor cells that close to the microvessels with decent lectin binding was observably lower than that of the microvessels with low lectin binding (Figure 6D,E). This finding indicated that the basal microvilli microvessels with limited lectin or CD31 binding in murine PCs have a strong nutrient trafficking ability, suggesting that lectin perfusion does not reflect the nutrient trafficking or perfusion of microvessels, especially the microvasculature with basal microvilli.

## 4 DISCUSSION

The hypomicrovasculature in PCs was reported to be poorly perfused, collapsed, and inefficient in nutrient trafficking.^4^, ^5^, ^6^ However, glucose and albumin, which depend on the facilitated‐trafficking mechanism, are easy to transport from the microcirculation to the PC milieu.^37^, ^38^ Protruding cellular projections are commonly used to increase surface area to intensify exchange, such as microvilli in the intestine and kidney.^10^, ^39^ Basal microvilli with nutrients trafficking apparatus present on the microvessels in PC. However, whether basal microvilli microvessels have an efficient perfusion and nutrient trafficking is unknown. By analyzing PC‐bearing GEMMs and PC patient data, we showed that the basal microvilli microvasculature is well perfused and efficient in nutrient trafficking, and might compensate the hypomicrovascularity in PC milieu.

The basal microvilli microvessels contain red blood cells.^11^ Here, we also noticed the basal microvilli quickly diminished when blood flow stopped. This implied that the growth of the basal microvilli depends on blood flow in microvessels. The observation of lectin dots in the inner surface of basal microvilli microvessels indicates that the basal microvilli microvessels are perfused. The interstitial pressure in PCs is high.^6^ The peripheral resistance increases the blood pressure in vessels. The microvessel density in PCs is close to 25% of that of a normal pancreas.^11^ The velocity of blood flow depends on the total cross‐sectional area of the blood vessels. If the total cross‐sectional area of the vessels decreases, the velocity of flow increases. PC microvasculature has a dense basal membrane and intact pericytes.^11^ These pieces of evidence implied that the velocity of microvasculature blood flow in PCs should be quicker than that of the pancreas. The observation of quicker blood trickle in the microvasculature of human PCs supports our hypothesis. The quicker trickle in the microvasculature might decrease the binding of perfused lectin or CD31 to its luminal receptors.

Oxygen, which depends on diffusion, is deficient in the PC milieu.^40^ However, glucose and albumin, which depend on facilitated trafficking, are high in PCs.^37^, ^41^, ^42^ Macropinocytosis is an efficient way to take up extracellular components and trafficking nutrients.^43^ Macropinocytosis exist in the basal microvilli.^11^ In TEM images, we observed a filopodia‐like structure in the luminal surface of PC microvessels. We observed that large 2‐NBDG bunches were preferentially located at the base of basal microvilli. The two pieces of evidence indicate that macropinocytosis might be a method of trafficking massive amounts of nutrients from the lumen of the basal microvilli microvessels to the tumor milieu. Of note, we also observed 2‐NBDG dots in the basal microvilli. Our recent data in more than 70 PC patient samples also showed that the abundance of basal microvilli is *bona‐fide* correlated with glucose uptake in tumors (unpublished data). Taken together, the basal microvilli microvessels in PCs might be the main segments in the microvasculature net where nutrient exchanges occur, and macropinocytosis might be the dominant way of exchanging nutrients or waste.

Collectively, our data showed that perfused labeling with lectin or CD31 is an efficient method that is equal to immunostaining to show the microcirculation in normal murine organs except kidney. However, perfusion with lectin and CD31 is inefficient for showing the microcirculation in PC and precursor lesions, especially basal microvilli microvessels, and does not reflect the nutrient trafficking status in the microvessels of PCs. Based on our observations, we argue that most microvessels in PCs are well perfused and efficient in nutrient trafficking, especially the basal microvilli microvessels, indicating that basal microvilli microvessels might be the major segment where nutrient and waste exchange occurs.

## CONFLICT OF INTEREST

All authors declare no competing financial interests.

## AUTHOR CONTRIBUTIONS

HS conceptualized the design. HS and LXM did animal experiment. XH, JG, JL, and WHL collected fresh samples. LXM supported this project. HS performed all staining, scanning, and analyzing data. HS wrote the paper. CJ obtained clinical videos of microcirculation and provided advices for interpreting data.

## Supporting information

Supplementary Material

Supplementary Material

Supplementary Material

## Data Availability

All data of this study are available upon special request to the corresponding authors.
