# Supplementary material for: The physiological characteristics of the basal microvilli microvessels in pancreatic cancers
Source: Cancer Med. 2020 Jun 2;9(15):5535–45. doi: 10.1002/cam4.3177 (PMC7402840; doi:10.1002/cam4.3177)
Supplement: Supplementary file 1 — Supplementary Material [file CAM4-9-5535-s001.doc]

**Supplementary information**

Supplementary figure


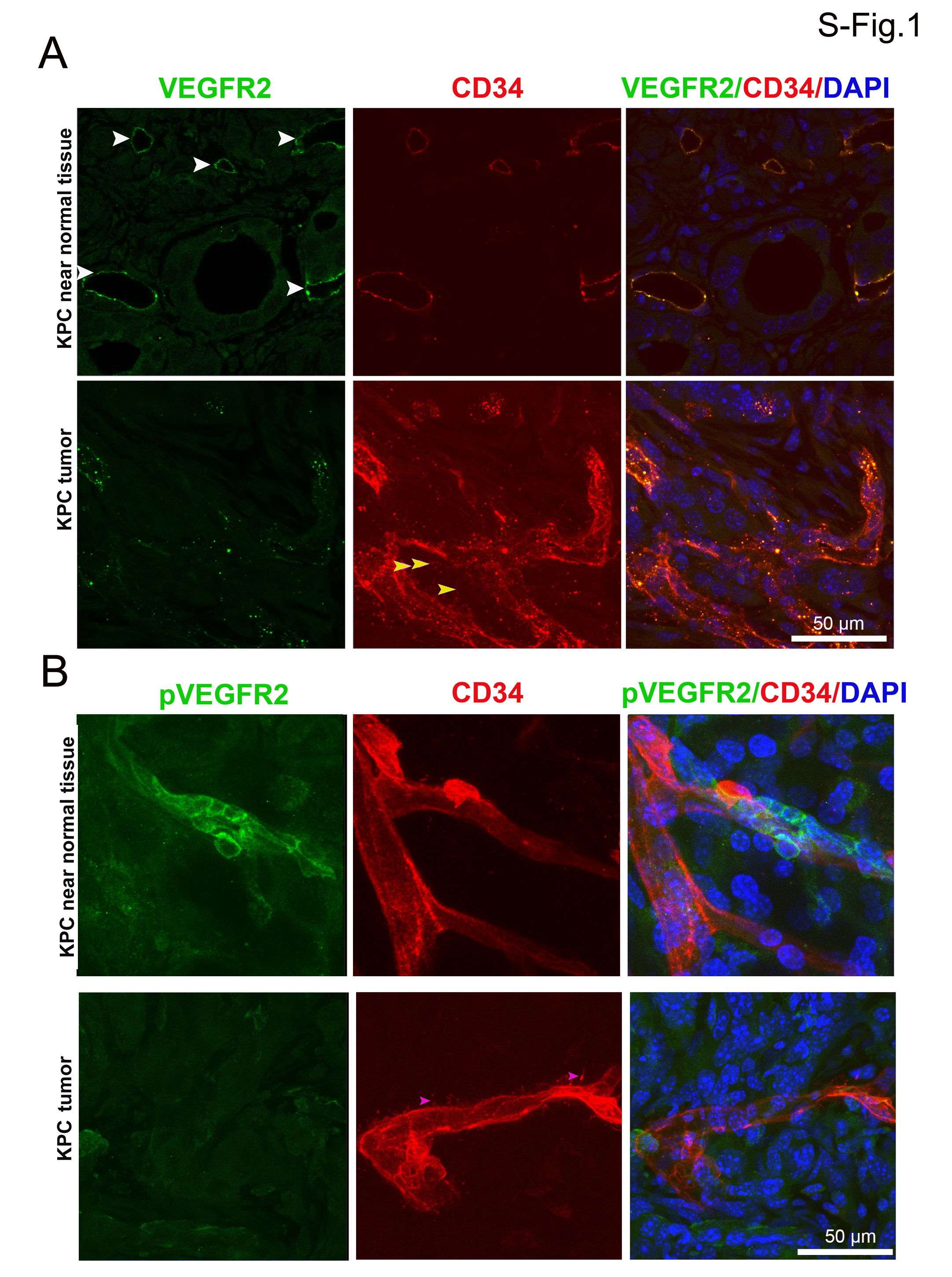


S-figure-1. The expression of VEGFR2 and phospho-VEGFR2^Y996^ in the PC and near-normal pancreas in KPC mice

A. Comparison of VEGFR2 expression patterns in the basal microvilli microvessels of the PC in KPC with that in the near-normal pancreatic tissue of KPC mouse (yellow arrows, basal microvilli).

B. Comparison of phospho-VEGFR2^Y996^ (pVEGFR2^Y996^) expression levels in the basal microvilli microvessels of the PC in KPC with that in the near-normal pancreatic tissue of KPC mouse (yellow arrows, basal microvilli). Mice, n=2.

**Supplementary video legends**

S-video 1 and S-video 2. The representative videos of the blood flow of the microcirculation in the healthy human pancreas and PC. Videos were recorded by probe-based laser confocal endomicroscopy.
